# Unusual presentation of meningococcal meningitis in the elderly and utility of CSF PCR testing

**DOI:** 10.1099/acmi.0.000158

**Published:** 2020-08-12

**Authors:** Elizabeth Ranson, Hannah Ship, Omai Garner, Shangxin Yang, Debika Bhattacharya

**Affiliations:** ^1^​ Division of Infectious Diseases, Department of Medicine, University of California, Los Angeles, USA; ^2^​ Clinical Microbiology Laboratory, Department of Pathology and Laboratory Medicine, UCLA Medical Center, Los Angeles, USA

**Keywords:** elderly meningitis, meningococcal meningitis, multiplex PCR, PCR test

## Abstract

We present an unusual case of a previously healthy 74-year-old man who presented with diffuse weakness, severe myalgias, petechial palmar rash and hypotension, but without fever, altered mental status, nuchal rigidity or headache, who was ultimately found through PCR testing to have meningococcal meningitis.

## Introduction

Despite advances in vaccination and treatment, bacterial meningitis remains a deadly disease [1]. The elderly are especially at risk, with higher rates of adverse complications and mortality rates of 20–25 % [2–5]. Because of the success of childhood vaccination programs, the disease burden of bacterial meningitis is now primarily borne by the elderly [2]. Moreover, in a disease in which prompt diagnosis and treatment are critical to minimizing morbidity and mortality [6], the elderly are especially vulnerable, as they are less likely to present with classic symptoms, leading to potential delay in diagnosis [3, 5, 7, 8]. While most patients present with at least two of the classic symptoms of fever, neck rigidity, altered mental status and headache, elderly patients are more likely to present with only one of these symptoms. However, it is extremely rare for any patient to present with none, as our patient did [9–11].

We present a case of a previously healthy 74-year-old man who presented with diffuse weakness, severe muscle aches, rash and hypotension, but without fever, altered mental status, neck rigidity or headache, and who was ultimately found to have meningococcal meningitis.

Given the high mortality of bacterial meningitis, examination of unusual presentations can contribute to better understanding of clinical presentations and lead to faster diagnosis and better outcomes. This case occurred in June 2016, during a larger local outbreak of *Neisseria meningitidis* in the Los Angeles area [12], but our patient did not present with typical clinical findings nor typical risk factors. Examination of his case may lead to improved diagnosis and public health interventions.

### Case report

A previously healthy 74-year-old man presented to the emergency room with 2–3 days of globus sensation, dysphagia, diffuse weakness, severe muscle aches and subjective fevers. His initial symptom was globus sensation, which progressed to difficulty swallowing solids and then liquids. Weakness and aches had also progressed to the point that he reported he was unable to walk. The day of admission, he tried to drink some water but had one episode of nonbloody nonbilious emesis. He denied nausea, headaches, changes in vision, stiff neck, altered mental status, cough, chills or sick contacts. At baseline he was fully independent, ran a successful business, and was monogamous with his husband of many years. He denied smoking, or using illicit drugs.

On presentation he was found to be afebrile with a temperature of 36.7 °C. His heart rate was 67 beats per minute and blood pressure 95/61 mmHg, which dropped to 65/48 mmHg within an hour of presentation. He was fully oriented and interactive, and his exam was notable for 4/5 weakness in bilateral upper and lower extensor and flexor muscles, severe total body myalgias, and a petechial rash on the palms and soles of his feet. His neck was supple, and the remainder of his neurological exam was within normal limits. Laboratory studies were notable for a platelet count of 139 000 μl^–1^ [normal range 143 000–398 000] (noted to be 229 000 2 weeks prior to presentation) and a leukocytosis of 17 020 μl^–1^ [normal range 416 000–995 000], 88 % neutrophils [normal range 40–60 %], concerning for bacterial infection. Additional laboratory studies showed an acute kidney injury with creatinine of 2.3 mg dl^–1^ (1.0 2 weeks prior), an elevated erythrocyte sedimentation rate of 46 mm h^–1^, creatinine kinase elevated at 197 U l^–1^, fibrinogen 561 mg dl^–1^, lactate 40 mg dl^–1^, HIV- 1/2 Ag/Ab negative, complement normal, and cortisol 54 mcg dl^–1^. C-reactive protein was found to be abnormal at 26.8 mg dl^–1^. His blood pressure responded to volume resuscitation with normal saline. The patient was started on vancomycin and piperacillin/tazobactam, and he was admitted to the intensive care unit. Blood cultures were then drawn and were sterile.

Then, 36 h after initial presentation and administration of antibiotic, the patient had a fever of 38.1 °C. MRI brain and cervical spine showed ‘layering restricted diffusion in the lateral ventricles bilaterally…[concerning for] layering pus in the setting of ventriculitis.’ Piperacillin /tazobactam was switched to meropenem, and voriconazole and ampicillin were added to his antimicrobial regimen. Attempts at beside lumbar puncture failed secondary to degenerative spinal disc disease, and lumbar puncture was finally performed on hospital day 4 under CT guidance. Cerebral spinal fluid (CSF) showed 3000 cells per cubic millimeter (cmm) red blood cells, 1654 cmm white blood cells (76 % neutrophils, 16 % monocytes, 8 % lymphocytes), 81 mg dl^–1^ glucose (serum glucose 152 mg dl^–1^) and 93 mg dl^–1^ protein. CSF Gram stain showed no bacteria with many white blood cells, and CSF culture was negative (in the setting of broad antimicrobial therapy for 4 days). On hospital day 6 the BioFire FilmArray PCR panel demonstrated *N. meningitidis *. The patient recovered well with no residual neurological deficits and was discharged home on hospital day to complete a 14-day course of ceftriaxone.

## Discussion

Given the high and swift mortality associated with bacterial meningitis, learning from unusual presentations may aid faster diagnosis in the future. This case illustrates several important clinical points, including an unusual presentation of meningitis in an elderly individual, the utility of PCR-based testing of CSF especially with prior antibiotic use, and the importance of meningococcal vaccination.

Although the sensitivity of the classic triad of fever, neck stiffness and altered mental status is less than 50 %, when headache is added as a diagnostic criterion the sensitivity of 2/4 symptoms rises to 99–100 % [9]. Fewer than 1 % of patients with bacterial meningitis present without any of these symptoms, as our patient did, even among the elderly [9, 10]. Of interest, our patient did present with two classic symptoms specific to invasive meningococcal disease: severe myalgias and petechial rash [9, 11].

Additionally, the identification of a specific pathogen hinged upon the BioFire FilmArray PCR panel. The FA Meningitis/Encephalitis Panel is a qualitative *in vitro* PCR assay that simultaneously tests for 14 common pathogens in meningitis and encephalitis [13].

In our case, likely because of delay in lumbar puncture, Gram stain and CSF culture were unrevealing. While CSF cell count and chemistry led to the diagnosis of bacterial meningitis, the identification of a specific pathogen using PCR not only led to targeted therapy but also directed important post-exposure prophylaxis (PEP) of close contacts.

While CSF culture remains the diagnostic gold standard for bacterial meningitis [14], the time to result (frequently more than 48 h) can prolong exposure to broad-spectrum antibiotics and delay PEP for close contacts when indicated. Moreover, the yield of CSF culture decreases quickly with the administration of antibiotics prior to lumbar puncture [15, 16]. Sterilization of CSF appears to be especially rapid with *N. meningitidis *. One study showed that one third of CSF samples with meningococcal infection were sterile within 1 h of antibiotic administration, and all samples were sterile within 2 h [17]. Even when meningitis is suspected at presentation, a delay of 2 h prior to lumbar puncture is common [18].

As an additional diagnostic tool, CSF Gram stain can rapidly and accurately identify a causative bacterium in 60–90 % of patients with community-acquired bacterial meningitis, with a specificity of >97 % [19]. However, the sensitivity of Gram stain depends on concentration of bacteria in CSF and the organism present [1, 20]. Most studies have shown that decrease in sensitivity of Gram stain after the administration of antibiotics is minimal, but, as with culture, the sensitivity drops more with *N. meningitidis* compared to other common pathogens, ranging from 30–89 % [1, 16].

PCR technology has become cheaper and faster, making it a more accessible clinical tool. PCR tests, through amplification of a specific genetic target of a micro-organism, is generally more sensitive than conventional culture-based methods and is not affected by the viability of the micro-organisms, especially those that are fastidious and hard to grow in the lab. One study used a latent class analysis model, and found the sensitivity and specificity estimates were: culture, 81.3 and 99.7 %; Gram stain, 98.2 and 98.7 %; and real-time PCR, 95.7 and 94.3 %, respectively. The sensitivity of Gram stain and PCR did not change significantly when antibiotics were also present in the CSF [21]. The wider utilization of the BioFire Meningoencephalitis (ME) Multiplex PCR assay, which was used in this case, had been shown by several studies to enhance the speed and accuracy of the diagnosis of CNS infections [22–24]. In a preclinical assessment study, compared to the standard care (singleplex PCR, culture, and Gram stain), the BioFire ME assay was found to have 92.9 and 91.9% positive and negative agreements, respectively [25]. In another multicenter evaluation of the BioFire ME assay, 100 % positive agreement was observed in 9/14 analytes compared to culture and singleplex PCR [26]. However, it should be noted that false-positive results can be common and the positive predictive value varies from 100 % to only 50 % [27]. The false-positive results happened more frequently with several high-prevalent viruses such as HSV-1, VZV and HHV-6, as well as Cryptococcus that is most likely due to environmental contamination [27, 28]. In one case, a tuberculous meningitis was misdiagnosed as HSV-1 encephalitis due to a false positive HSV-1 result by the BioFire ME assay [27]. False-negative results can also occur. In one study, the BioFire ME assay missed 2/38 enterovirus, 1/15 parechovirus, and 2/4 HSV-1 [29]. These studies pointed out both advantages and limitations of the BioFire ME assay. Clinicians should be aware of these issues and not base diagnosis on PCR results alone, but in the context of the clinical pictures and other test results, including Gram-stain tests, to ensure reliable determination of the infecting species. Additionally, BioFire does not differentiate serotypes for many organisms, which may be important, especially in outbreaks.’

In addition to increasing awareness of unusual presentation of meningococcal meningitis in the elderly, this case also illustrates the benefit of expanded vaccination. This patient presented in Los Angeles during a known outbreak of *N. meningitidis* among young men who have sex with men (MSM). Although our patient was MSM, he endorsed a long-term, monogamous marriage, and denied what are considered typical risk factors such as bars or dating apps, and he had not received any meningococcal vaccinations. In 2016, Los Angeles County reported 17 cases of invasive meningococcal disease (up from 12 in 2015); 9 (60 %) in MSM, with a median age of 32 (range 16–76) [12]. The larger Los Angeles area reported 35 cases [12]. Outbreaks are of especial concern because, although only approximately 2 % of total meningococcal disease occurs as part of outbreaks, mortality in outbreaks is twice as high as in sporadic cases [30]. It is unclear why MSM are at higher risk than the general population, but epidemiologic studies (from the 1980s) found high colonization of the oropharynx, rectum and urethra with *N. meningitidis* among MSM [31]. Based on the demographics of the current outbreak, Los Angeles County has now broadened its vaccination recommendations to all MSM [32, 33].

This unusual presentation of *N. meningitidis* highlights the utility of PCR-based testing of CSF, and advancements in reliable, rapid detection.
